# Sequential Metabolism of 7-Dehydrocholesterol to Steroidal 5,7-Dienes in Adrenal Glands and Its Biological Implication in the Skin

**DOI:** 10.1371/journal.pone.0004309

**Published:** 2009-02-03

**Authors:** Andrzej T. Slominski, Michal A. Zmijewski, Igor Semak, Trevor Sweatman, Zorica Janjetovic, Wei Li, Jordan K. Zjawiony, Robert C. Tuckey

**Affiliations:** 1 Department of Pathology, University of Tennessee Health Science Center, Memphis, Tennessee, United States of America; 2 Department of Pharmacology, University of Tennessee Health Science Center, Memphis, Tennessee, United States of America; 3 Department of Biochemistry, Belarus State University, Minsk, Belarus; 4 Department of Pharmaceutical Sciences, University of Tennessee Health Science Center, Memphis, Tennessee, United States of America; 5 Department of Pharmacognosy, University of Mississippi, Oxford, Mississippi, United States of America; 6 School of Biomedical, Biomolecular and Chemical Sciences, The University of Western Australia, Crawley, Western Australia, Australia; University of Helsinki, Finland

## Abstract

Since P450scc transforms 7-dehydrocholesterol (7DHC) to 7-dehydropregnenolone (7DHP) *in vitro*, we investigated sequential 7DHC metabolism by adrenal glands *ex vivo*. There was a rapid, time- and dose-dependent metabolism of 7DHC by adrenals from rats, pigs, rabbits and dogs with production of more polar 5,7-dienes as detected by RP-HPLC. Based on retention time (RT), UV spectra and mass spectrometry, we identified the major products common to all tested species as 7DHP, 22-hydroxy-7DHC and 20,22-dihydroxy-7DHC. The involvement of P450scc in adrenal metabolic transformation was confirmed by the inhibition of this process by DL-aminoglutethimide. The metabolism of 7DHC with subsequent production of 7DHP was stimulated by forscolin indicating involvement of cAMP dependent pathways. Additional minor products of 7DHC metabolism that were more polar than 7DHP were identified as 17-hydroxy-7DHP (in pig adrenals but not those of rats) and as pregna-4,7-diene-3,20-dione (7-dehydroprogesterone). Both products represented the major identifiable products of 7DHP metabolism in adrenal glands. Studies with purified enzymes show that StAR protein likely transports 7DHC to the inner mitochondrial membrane, that 7DHC can compete effectively with cholesterol for the substrate binding site on P450scc and that the catalytic efficiency of 3βHSD for 7DHP (V_m_/K_m_) is 40% of that for pregnenolone. Skin mitochondria are capable of transforming 7DHC to 7DHP and the 7DHP is metabolized further by skin extracts. Finally, 7DHP, its photoderivative 20-oxopregnacalciferol, and pregnenolone exhibited biological activity in skin cells including inhibition of proliferation of epidermal keratinocytes and melanocytes, and melanoma cells. These findings define a novel steroidogenic pathway: 7DHC→22(OH)7DHC→20,22(OH)_2_7DHC→7DHP, with potential further metabolism of 7DHP mediated by 3βHSD or CYP17, depending on mammalian species. The 5–7 dienal intermediates of the pathway can be a source of biologically active vitamin D3 derivatives after delivery to or production in the skin, an organ intermittently exposed to solar radiation.

## Introduction

For decades it has been assumed that in mammalian systems all major steroidogenic pathways, including the chemical structures of final products and intermediates, are well established. These pathways start with cytochrome P450scc mediated production of pregnenolone, a precursor to all steroids [1], [2]. P450scc (product of the CYP11A1 locus) is a mitochondrial enzyme that catalyzes two successive hydroxylations of the cholesterol side chain at positions 22 and 20, followed by its cleavage to produce pregnenolone and isocaproic aldehyde [3], [4]. Electrons for the reactions are provided by NADPH via adrenodoxin reductase and adrenodoxin [5], [6]. P450scc is predominantly expressed in adrenals and gonads which produce steroid hormones for systemic use, and is expressed at a lower level in the placenta. Very low expression of P450scc has also been detected in brain [7], kidney [8], gut [9], and skin [10]–[12], indicating production of pregnenolone at these sites for local use.

Most recently it has been uncovered that the same enzyme, P450scc, cleaves the side chain of 7-dehydrocholesterol (7DHC), a direct precursor to cholesterol to produce 7-dehydropregnenolone (7DHP) [10], [13]. The efficiency of this process is the same as for cholesterol conversion to pregnenolone [10]. This suggests that P450scc action on 7DHC can start a novel metabolic pathway for production of steroids with an unsaturated B ring [10]. 7-DHC contains an unsaturated B ring (two double bonds in position 5 and 7) that is reduced by delta-7 reductase to produce cholesterol [14], [15]. Defects in this enzyme leads to reduced or absent 7DHC transformation to cholesterol with accumulation of steroidal 5,7-dienes, well described in the Smith Lemli Opitz Syndrome (SLOS) [16]–[18]. The latter suggests an *in vivo* action of P450scc on 7DHC, at least under the pathological condition of SLOS.

Skin is an example of an organ with a low steroidogenic potential [10], [19]. However, it accumulates significant amounts of 7DHC which is localized to the plasma membrane of basal epidermal keratinocytes, where it undergoes photolysis of the B ring upon absorption of photons of ultraviolet light B (wavelength 280–320 nm) to form previtamin D3, which then undergoes internal rearrangement to form vitamin D3 [19]–[22]. Interestingly, vitamin D3 as well as plant derived ergosterol or vitamin D2, are also metabolized by P450scc without cleavage of the side chain in reactions that generate mono-, di-, tri-hydroxy products [12], [13], [23]–[25], some of which show biological activity in skin cells cultured *in vitro* [12], [23], [26].

Thus, it has become crucial to test whether organs expressing P450scc can generate 5,7-diene steroids and to define whether they are biologically active. To test this hypothesis we have incubated fragments of adrenal glands (an example of a tissue with high P450scc activity) from different mammalian species with 7DHC and tested its metabolism to 7DHP and it derivatives. The capability of skin extracts (example of a tissue with low P450scc activity) to metabolize 7DHC and 7DHP was also tested. To establish the relative biological activity of the intermediates of the pathway we used skin cells to test the effects of products of P450scc action on cholesterol and 7DHC, namely, pregnenolone and 7DHP, as well as the product of UV mediated photolysis of 7DHP, 20-oxopregnacalciferol (pD3).

## Results and Discussion

### 1. Metabolism of 7DHC in the adrenal glands ex vivo

**Figure 1:**
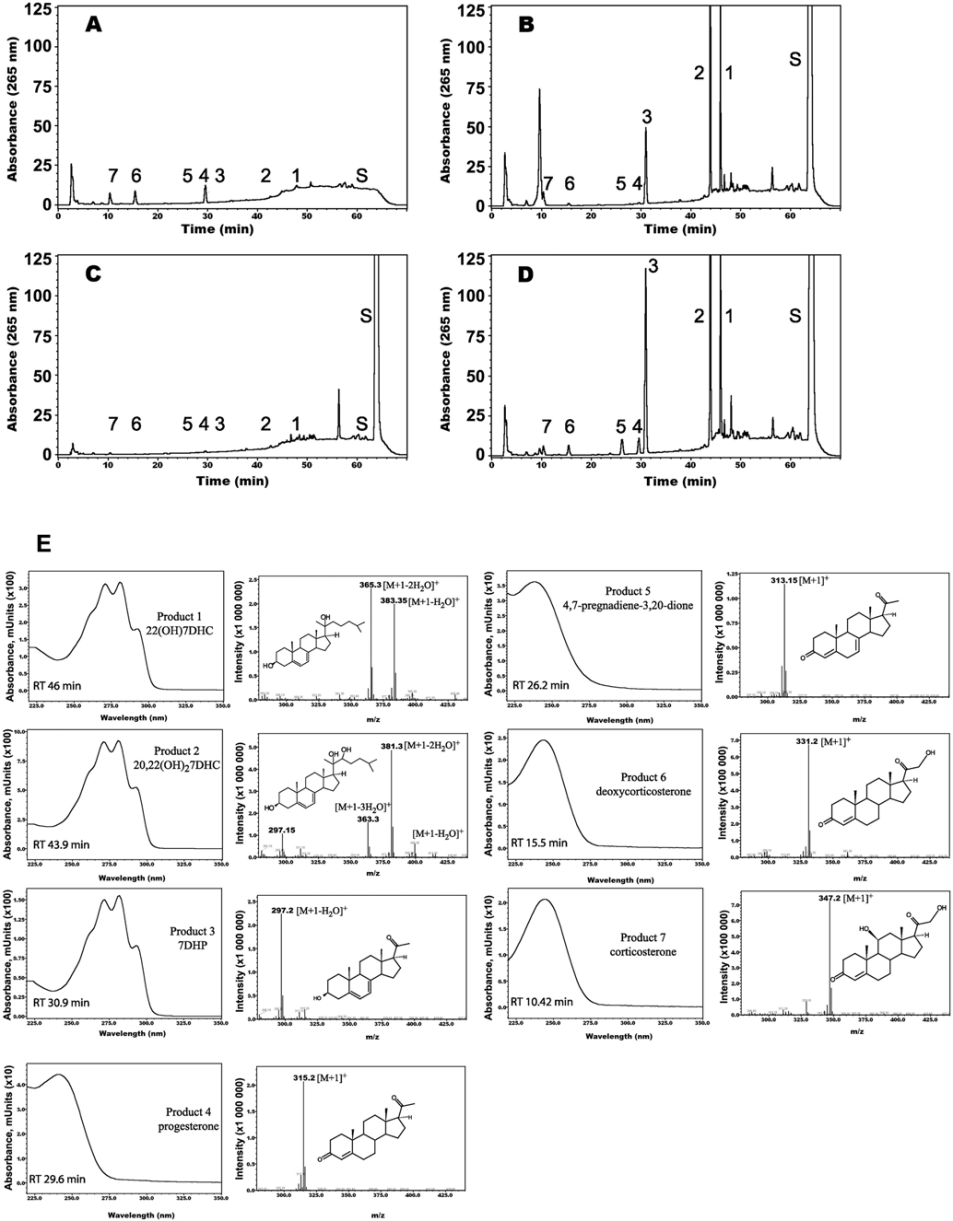
7DHC metabolism by rat adrenal glands. Finely cut fragments of rat adrenal glands were incubated in the presence (B–D) or absence (A) of 7DHC (0.5 mM). Boiled adrenal fragments (C) served as an additional negative control. Panel B shows results from an incubation which included trilostane, a 3βHSD inhibitor. Steroids were extracted with methylene chloride and subjected to LC/MC and UV spectra analyses. Panels A–D identifies 7 main products. Product 3 had an identical retention time to 7DHP standard. Panel E shows UV spectra, MS and the predicted structures of the 7 metabolites.

**Figure 2:**
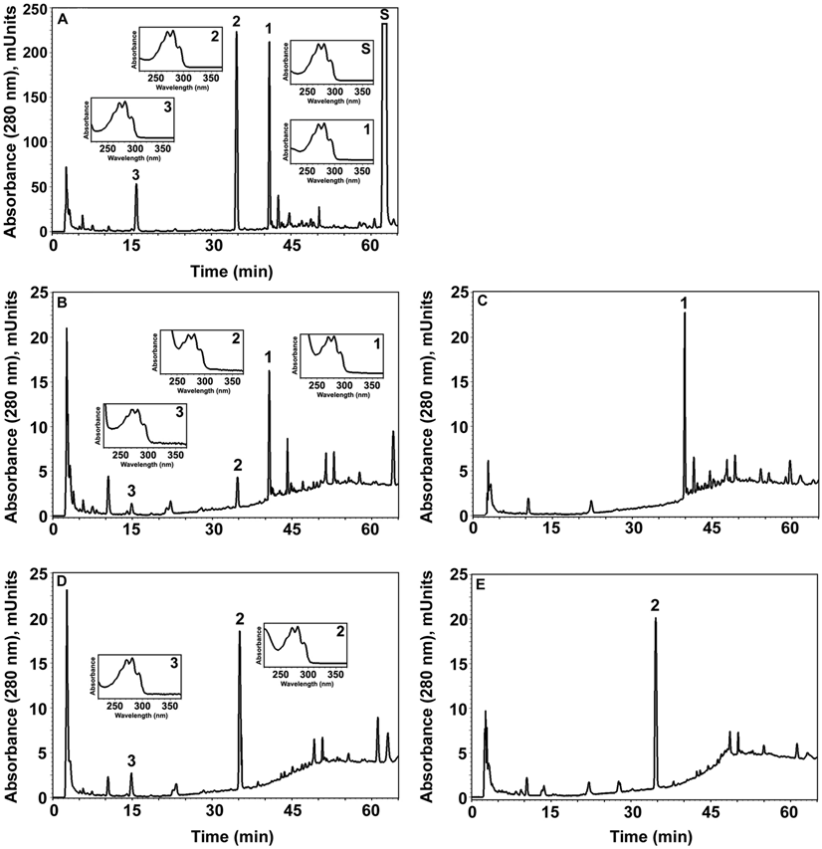
Precursor-product relationship between 7DHC, hydroxy-7DHC, dihydroxy-7DHC and 7DHP defined by their sequential metabolism by rat adrenal glands. A. Chromatogram showing metabolism of 7DHC(S) by adrenal glands. The hydroxy-7DHC (1) and dihydroxy-7DHC (2) products were collected from the HPLC and tested as substrates (see B–E). Product 3 was identified as 7-DHP based on RT, mass and UV spectra. B. Incubation of hydroxy-7DHC (1) with adrenal glands generated dihydroxy-7DHC with RT 34.8 min (2) and 7DHP with RT 15.8 min (3). C. Boiled adrenal glands served as a control for B. D. Incubation of dihydroxy-7DHC (2) with adrenal glands produced 7DHP; note the lack of S and product 1. E. Negative control for D (boiled adrenal glands ). Inserts: UV spectra of compounds 1–3.

We incubated minced rat adrenal glands with (test) or without 7DHC (negative control) as described in the Materials and Methods. A second negative control comprised boiled adrenal glands incubated with 7DHC. The samples were extracted with methylene chloride and analyzed on an LC–MS QP8000a equipped with diode array and single quadrupole mass-spectrometric detectors. Results of a representative experiment (Fig. 1) show that 7DHC is metabolized by adrenal glands to four specific products represented by three major peaks (peaks 1–3) and one minor peak (peak 5, Fig. 1D). Peaks 4, 6 and 7 correspond to products of endogenous cholesterol metabolism as they are present in the control (Fig. 1A) and are identified as progesterone, deoxycorticosterone and corticosterone, based on their mass and UV spectra as well as their retention times (RT) compared to authentic standards (Fig. 1 E). Using the same identification criteria (Fig. 1 E), product 3 is identified as 7DHP, and products 1 and 2 tentatively as 22-hydroxy-7DHC (22(OH)7DHC), 20,22-dihydroxy-7DHC (20,22(OH)_2_7DHC), respectively. Product 5 is defined as 4,7-pregandien-3,20-dione (7-dehydroprogesterone), which is consistent with m/z = 313 for [M+1]^+^ (real mass of 312), UV λmax of 238, and its disappearance after incubation with trilostate (T), an inhibitor of 3β-hydroxysteroid dehydrogenase (3βHSD) (Fig. 1 B, E). To establish a precursor-product relationship between compounds 1–3 we performed an additional experiment where purified products 1 and 2 were individually incubated with rat adrenal glands (Fig 2). This shows that there is sequential transformation of product 1 to the more polar 5,7- diene (product 2) and 7DHP, and the sole conversion of product 2 to 7DHP. Taking into consideration that the initial hydroxylation of the cholesterol side chain occurs at C22 [27] and given that 7DHC and cholesterol metabolism by P450scc are very similar [10], we defined product 1 as 22(OH)7DHC and product 2 as (20,22(OH)_2_7DHC).

**Figure 3:**
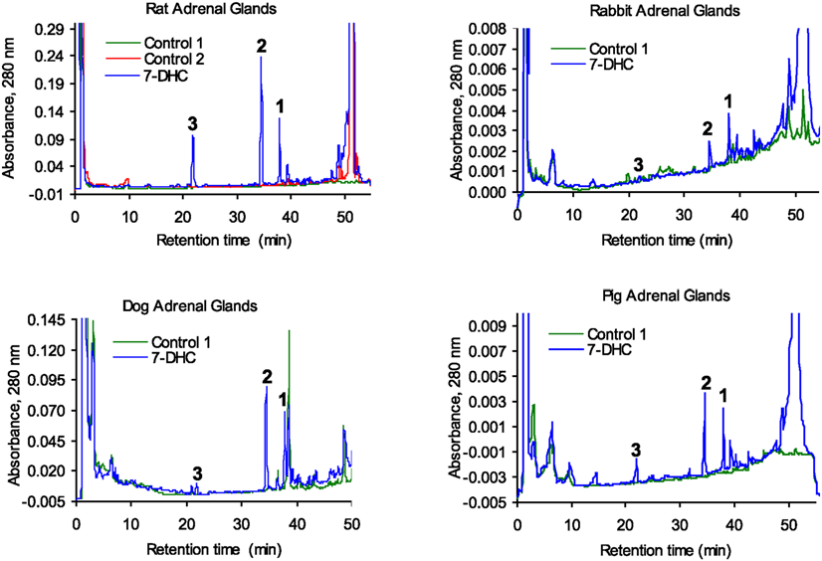
Metabolism of 7-DHC by adrenal glands of different mammalian species. Finely cut fragments of rat (A), rabbit (B), dog (C) and pig (D) adrenals were incubated in the presence (blue) or absence (green) of 7DHC (S). Boiled adrenal fragments served as an additional negative control for the rat (red). The three major products common to all animals studied are numbered 1 to 3 on the chromatograms. Product 3 had an identical retention time, mass, and UV spectra to 7DHP standard.

**Figure 4:**
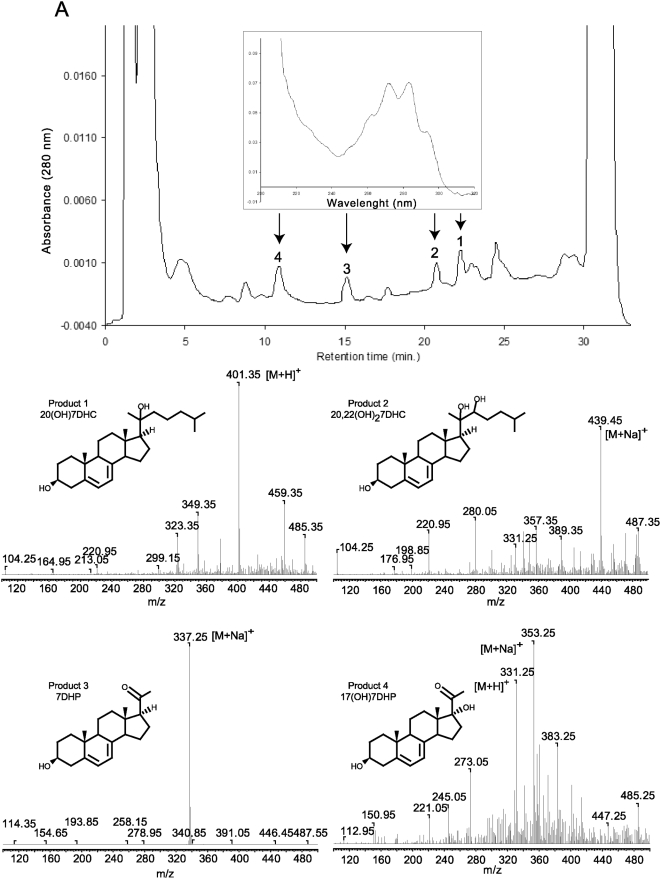
Metabolism of 7DHC by pig adrenal glands. Fragments of pig adrenal glands were incubated with 0.5 mM 7DHC. Steroids were extracted with methylene chloride and subjected to HPLC and UV spectral analyses. The UV spectrum for all peaks is the same (see arrows). The mass spectra of the collected peaks 1–4 were obtained by direct injection into the MS module of the Bruker Esquire-LC/MS Spectrometer.

Next we analyzed whether similar metabolism of 7DHC occurs in other mammalian species. Briefly, minced adrenal glands from rats (positive control), rabbits, dogs or pigs were incubated with or without 0.5 mM 7DHC and processed as described above. In these experiments the extracted samples were analyzed with a RP-HPLC Waters dual pump chromatography system equipped with a photodiode array. Figure 3 shows that adrenal glands from rabbits, dogs and pigs metabolize 7DHC to three major 5,7-diene products (UV λmax 261, 271, 281, 293), identified from rat samples as 7DHP, 22(OH)7DHC and 20,22(OH)_2_7DHC. These experiments also show the production of more polar compounds, one with RT 21 min (most likely 7-dehydroprogesterone as defined for compound with RT of 26.2 min in experiment presented in Fig. 1E and another which was a 5,7-diene with a short RT of 14.7 min seen in rabbit and pig samples (Fig. 3). The identity of the 7DHP was determined by its identical RT (22 min) with 7DHP synthetic standard. The production of 7DHP was time- and dose- dependent and dependent on the amount of tissue used. Separate LC/MS mass spectrometry analyses of the pig adrenal samples were performed on a Bruker Esquire-LC/MS Spectrometer equipped with an electrospray ionization source (ESI) and showed the expected real masses for monohydroxy-7DHC (1), dihydroxy-7DHC (2), 7DHP (3) and 17-hydroxy-7DHP (4) (Fig. 4). The collected peaks had the expected UV spectra for 5,7-dienes, and peaks 3 and 4 had identical retention times to 7DHP and 17(OH)7DHP standards. We thus conclude that mammalian adrenal glands metabolize 7DHC in the sequence 7DHC→22(OH)7DHC→20,22(OH)_2_7DHC→7DHP, with potential further metabolism of the 7DHP by 3βHSD or CYP17, depending on the mammalian species. The detection of 17(OH)7DHP in pig adrenals but not in those of rats is consistent with expression of CYP17 in the former but not latter species [28], [29].

**Figure 5:**
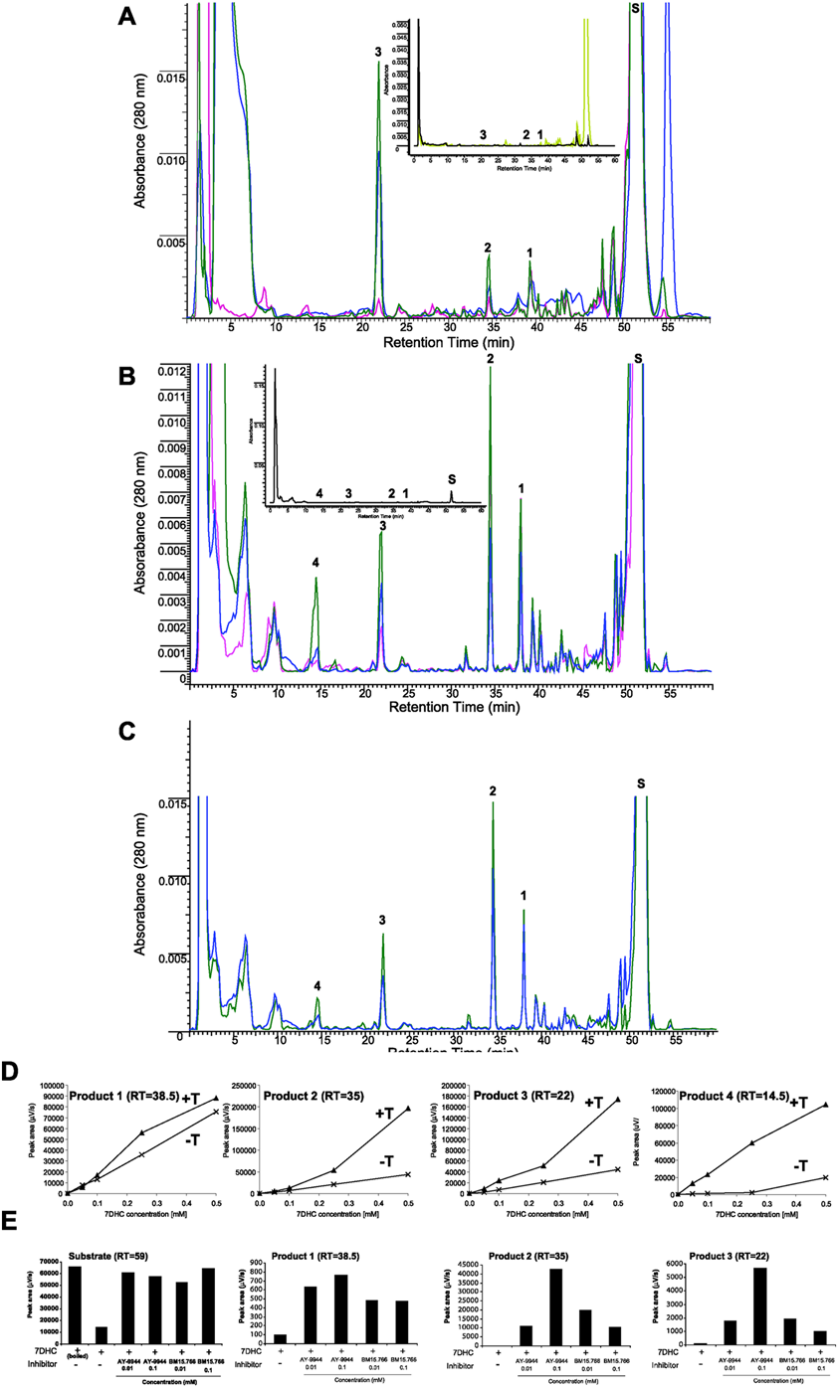
Metabolism of 7DHC by adrenal glands in the presence of DL-aminoglutethimide (AGT), trilostane, forscolin and Δ7-reductase inhibitors. A. For rat adrenals transformation of 7DHC (S) to 7DHP (3) was dramatically inhibited by AGT (red) compared to the incubation with 7DHC alone (blue), while accumulation of 22(OH)7DHC (1), 20,22(OH)_2_7DHC (2) and 7DHP (3) was enhanced by trilostane (green). Control incubations performed with boiled adrenal fragments are shown in the inset. Incubations were carried out for 18 h. B. For pig adrenals AGT also inhibited 7DHC metabolism (red) compared to the incubation with 7DHC alone (blue), while trilostane (green) enhanced accumulation of 22(OH)7DHC (1), 20,22(OH)_2_7DHC (2), 7DHP (3) and 17(OH)7DHP (4). Adrenal fragments were incubated for 6 h and controls (inset) were incubated without the substrate. C. For pig adrenals 0.1 mM forscolin (green) stimulated 7DHC metabolism. The labeling and the experimental conditions are the same as in B. D. Fragments of pig adrenal glands were incubated with increasing doses of 7DHC in the presence (T+) or absence (T−) of 0.1 mM trilostane. The chromatograms were processed as described above. Trilostane (T) enhanced accumulation of 22(OH)7DHC (1), 20,22(OH)_2_7DHC (2), 7DHP (3) and 17(OH)7DHP (4) as a function of 7DHC concentration. E. Fragments of rat adrenal glands were incubated with 7DHC (0.5 mM) in presence of AY-9944 or BM15.766 (10 µM or 100 µM) and analyzed by RP-HPLC (see A for peak identification). The chromatograms were processed using ACDLabs software and relative areas for peaks identified above were calculated. Δ7-reductase inhibitors enhanced production of 7DHC metabolites.

The sequential metabolism of 7DHC to 7DHP in rat and pig adrenal glands, and to 17(OH)7DHP in pig, was inhibited by DL-aminoglutethimide (AGT, an inhibitor of P450scc) and stimulated by forscolin (Fig. 5A–C). The former demonstrates that this metabolism is dependent on P450scc activity [30], while the latter indicates positive regulation by cAMP, as it is in case of the classical steroidogenic pathway that starts from the cholesterol [1], [31]. Furthermore, the accumulation of the above products was enhanced by trilostane (an inhibitor of 3βHSD) and by AY-9944 and BM15.766 (inhibitors of Δ^7^-reductase)(Fig. 5D,E). Thus the inhibition of 3βHSD with resulting accumulation of steroidal 5,7- diene intermediates (including hydroxy-7DHP) demonstrates that 7DHP enters the Δ^4^ steroidogenic pathway. This is further supported by detection of steroidal 4,7-dienes in SLOS patients [32], [33]. The increased accumulation of the 7DHC precursor and steroidal 5,7- diene intermediates after inhibition of Δ^7^-reductase demonstrate that the unsaturated B ring of 7DHC (and potentially of its metabolites) is reduced in the adrenal to generate cholesterol and the expected steroids. However, the relative efficiency of this catalytic reduction of the 7–8 double bond is very low as demonstrated in Fig. 1.

**Figure 6:**
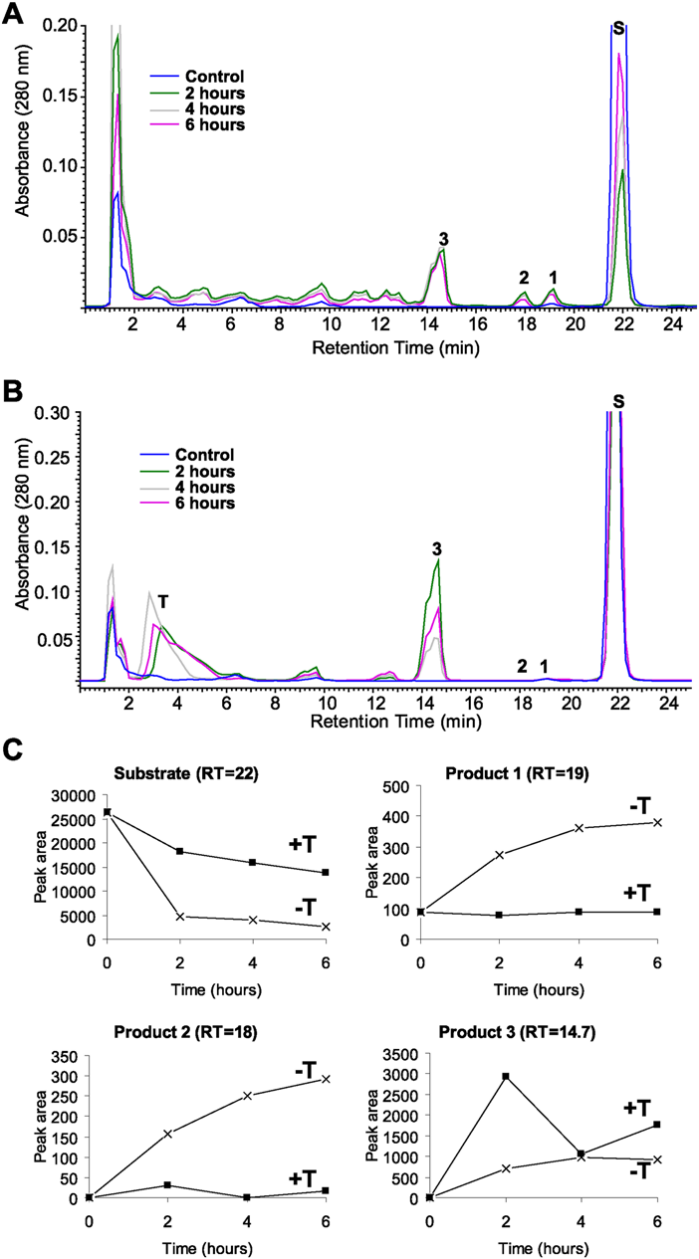
Time-dependent metabolism of 7DHP is modified by trilostane (T). Fragments of the pig adrenal glands were incubated with 7DHP (S; 0.5 mM) in the absence (A) or presence (B) of trilostane (0.1 mM) and analyzed by RP-HPLC. Boiled adrenal fragments were used as a negative control (blue). The major products of 7DHP metabolism affected by trilostane are marked by numbers. Time-dependent changes in the relative concentration of the identified products in presence (T+) or absence (T-) of trilostane were calculated using ACDLabs software and are presented in C.

**Figure 7:**
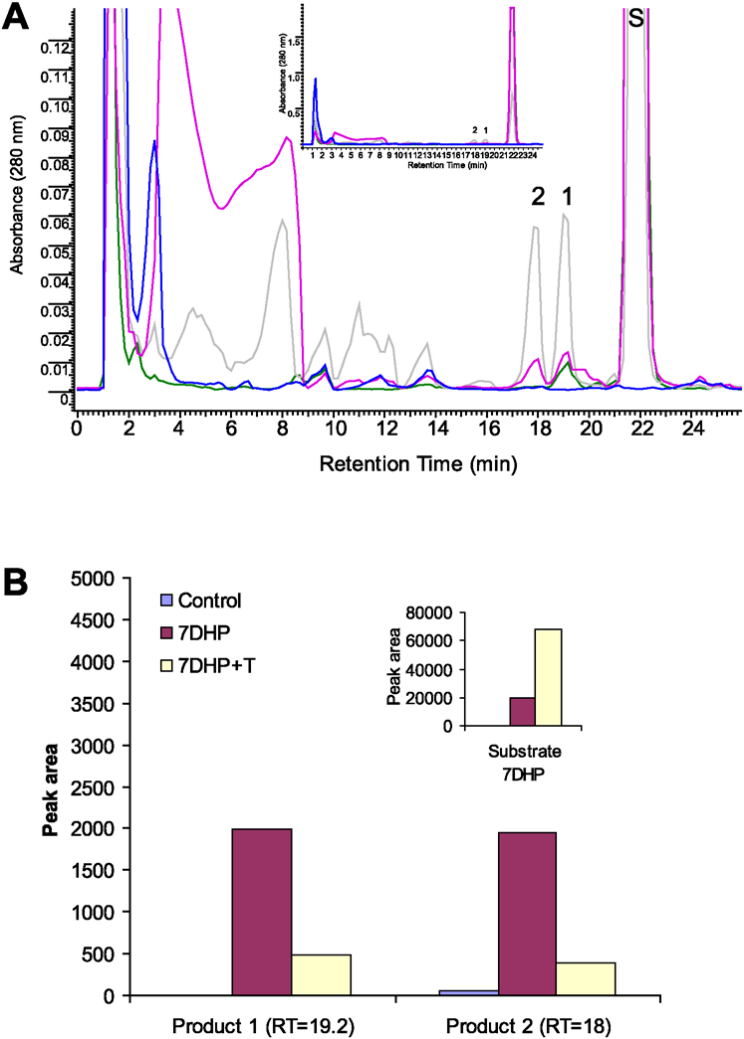
Trilostane (T) inhibits and modifies 7DHP (S) metabolism by rat adrenal glands. A. Fragments of rat adrenal glands were incubated with 7DHP (0.5 mM) in the absence (pink) or presence (gray) of trilostane (0.1 mM) and samples analyzed by RP-HPLC. Boiled adrenal fragments (green) or incubations without the substrate (blue) were used as negative controls. B. The relative concentrations of the identified products affected by trilostane (T). The insert shows inhibition of 7DHP consumption by trilostane. Control marked by blue represents or incubations without the substrate.

**Figure 8:**
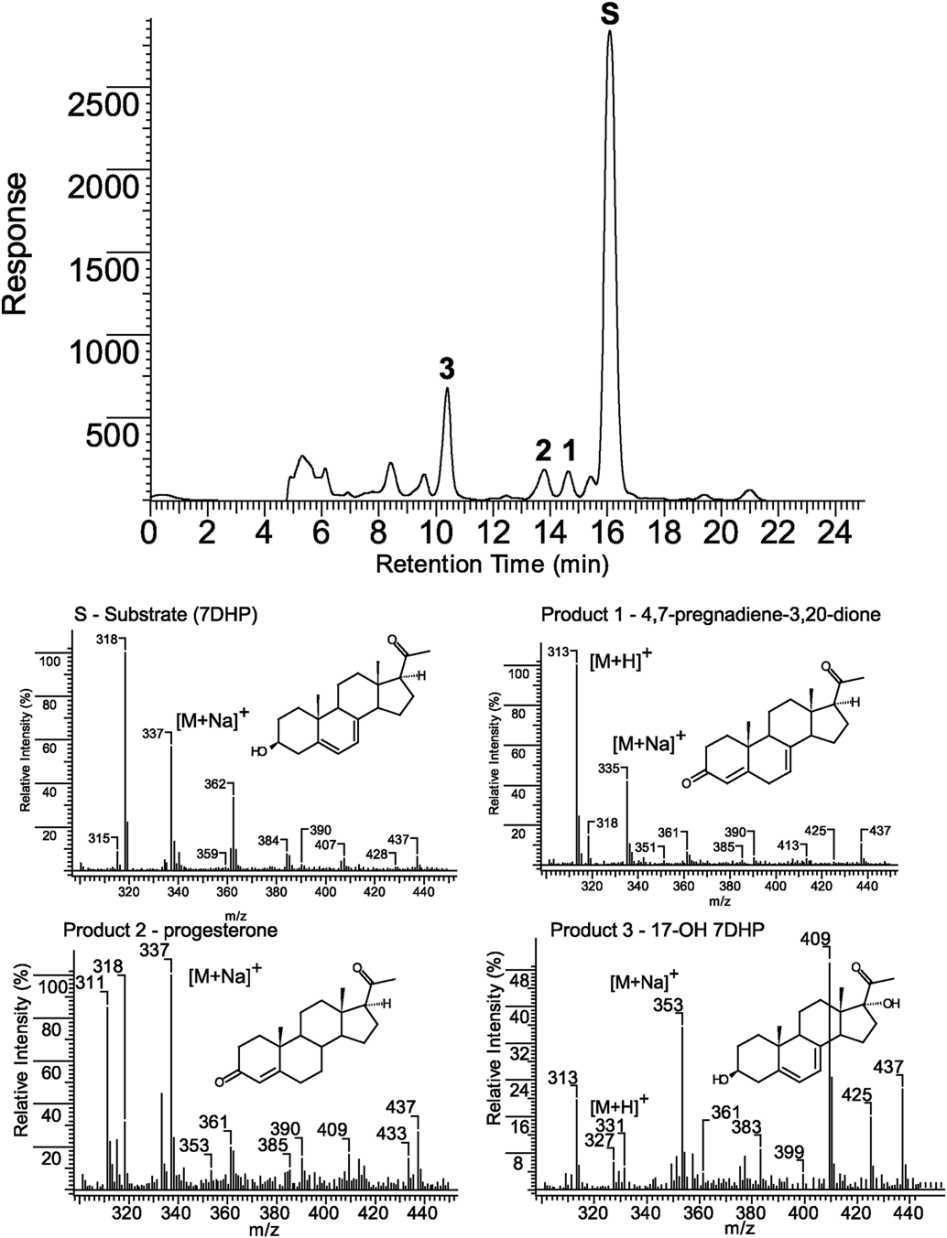
LC/MS identification of the products of 7DHP metabolism by pig adrenals. Collected fraction from RT-HPLC experiment were analyzed using the Bruker Esquire-LC/MS Spectrometer as described in Materials and Methods. Numbers correspond to the products identified in Figs 6 and 7.

**Figure 9:**
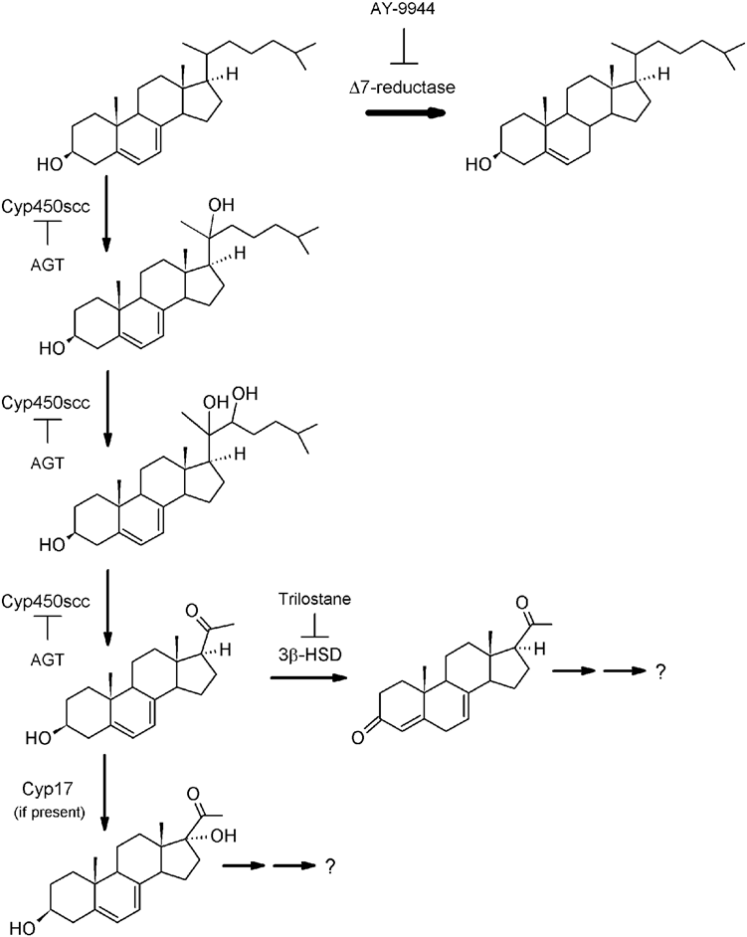
Proposed pathways for the *in vivo* metabolism of 7DHC in the mammalian adrenal gland.

To better define the later steps of the above metabolic pathway, we used 7DHP as the substrate. Incubation of 7DHP with minced adrenal glands resulted in time-dependent generation of more polar products (Fig. 6A and 7A). Production of the compounds corresponding to peaks with RT of 19.2 min (1) and 18 min (2) was inhibited by trilostane (Figs. 6B and 7), while the amount of product with RT of 14.7 min (3), present only in the pig, was increased (Fig. 6B, C). Product 3 had UV λmax (261,270,281,290 nm) and identical RT to 17(OH)7DHP. The LC/MS analysis performed with pig adrenal gland samples further confirmed that product 3 is indeed 17(OH)7DHP, and helped us assign products 1 and 2 as 7-dehydroprogesterone and progesterone, respectively (Fig. 8 and cf. Figs. 1, 6 and 7). These results further strengthen our proposal that *in vivo* sequential metabolism of 7DHC in adrenal glands is initiated by P450scc, involves 3βHSD and, depending on the species, CYP17 (Fig. 9). Importantly, this model provides additional proof that several metabolites identified in tissues and body fluids of SLOS patients [16], [32], [33] can indeed be of adrenal origin.

### 2. Experiments with purified enzymes

**Figure 10:**
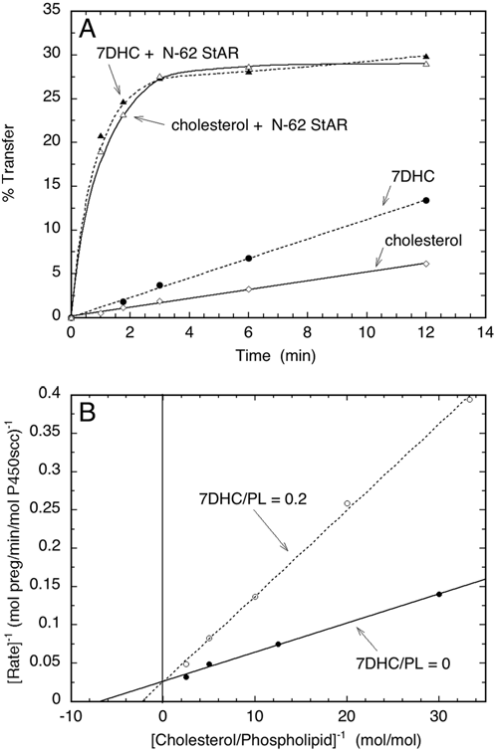
Stimulation of 7DHC transfer between membranes and the inhibition of the side chain cleavage of [cholesterol by 7DHC. A. To study 7DHC transfer between membranes by N-62 StAR protein cholesterol or 7DHC was placed in donor vesicles at a molar ratio to phospholipid of 0.2. The transfer of the sterol to acceptor vesicles at 35°C was measured in the presence or absence of 5 µM N-62 StAR protein. B. Inhibition of the side chain cleavage of [4-^14^C]cholesterol by 7DHC was determined with substrate and cytochrome P450scc incorporated into phospholipid (PL) vesicles prepared from dioleoyl phosphatidylcholine containing ardiolipin. As indicated in the figure, 7DHC was included in the membrane at a molar ratio to phospholipid of 0.2. The rate of side chain cleavage of [4-^14^C]cholesterol was determined from the amount of [4-^14^C]pregnenolone formed.

To further define the ability of steroidogenic enzymes in the adrenal cortex to metabolize 7DHC, additional studies were carried out with purified enzymes (Fig. 10). Metabolism of 7DHC by P450scc requires delivery of the 7DHC to the inner mitochondrial membrane where the P450scc is located [2]. Since cholesterol delivery to P450scc in the adrenal cortex and gonads is rate limiting for steroid synthesis and is mediated by the StAR protein [34], we tested the ability of recombinant N-62 StAR protein to transport 7DHC between phospholipid membranes. 7DHC displayed a basal rate of exchange between the membranes of phospholipid vesicles twice that for cholesterol. N-62 StAR protein (5 µM) stimulated the rate of 7DHC transfer by at least 20 fold, with the initial rate being too rapid to measure accurately (Fig. 10A). The rate of transfer of 7DHC by N-62 StAR protein was comparable to rate of cholesterol transfer by this carrier. Thus the StAR protein provides a likely carrier for transport of 7DHC to the inner mitochondrial membrane of the adrenal cortex for its metabolism by P450scc.

Once at the site of P450scc in the inner mitochondrial membrane, 7DHC must compete with cholesterol to be metabolized. Since both bovine and human enzymes display similar k_cat_/K_m_ values for 7DHC and cholesterol, close competition between these substrates would be expected [10]. To test this we examined the ability of unlabelled 7DHC to compete with, and thus inhibit, the conversion of [4-^14^C]cholesterol to [4-^14^C]pregnenolone by purified bovine P450scc incorporated into phospholipid vesicles (Fig. 10B). 7DHC at a molar ratio to phospholipid of 0.2 proved to be a competitive inhibitor of the side chain cleavage of cholesterol, increasing the K_m_ 3-fold without affecting V_max_. The calculated K_I_ for 7DHC (0.1 mol 7DHC/mol phospholipid was lower than the K_m_ for cholesterol (0.14 mol /mol phospholipid), therefore 7DHC can compete effectively with cholesterol for the substrate binding site on P450scc.

To determine the rate of metabolism of 7DHP by 3βHSD, we partially purified the type 1 enzyme from the human placenta. This enabled the activity of 3βHSD to be measured using 7DHP as substrate from the rate of NAD^+^ reduction. Specificity of the reaction for 3βHSD was demonstrated by the complete inhibition of NAD^+^ reduction by 8 µM cyanoketone (like trilostane, a 3βHSD inhibitor [35], [36]). K_m_ values for pregnenolone and 7DHP were 9.1 µM and 29.3 µM, respectively, while V_max_ values were 0.34 µmol/min/mg protein and 0.44 µmol/min/mg protein, respectively. Thus the catalytic efficiency of 3βHSD for 7DHP (V_m_/K_m_) is 40% of that for pregnenolone.

### 3. Biological activity of 7DHP, pregnenolone and 20-oxopregnacalciferol (pD3) in skin cells

**Figure 11:**
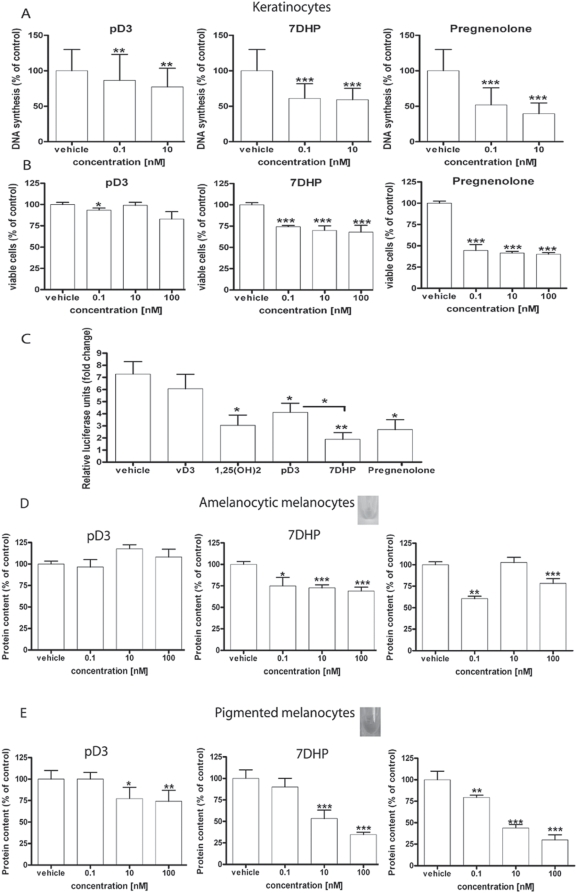
The effect of 7DHP, pregnacholecalciferol (pD3) and pregnenolone on normal immortalized epidermal HaCaT keratinocytes and PIG1 melanocytes. A and B. Inhibition of HaCaT cell proliferation as measured by [^3^H]-thymidine incorporation (A) and MTT (B) assays after 48 h and 72 h of treatment, respectively. C. Inhibition of NFκB activity in HaCaT keratinocytes 24 h after transfection with luciferase construct NFκB-Luc. The cells were treated for 24 h with 100 nM vitamin D3 (vD3), 1,25(OH)_2_D3, 7DHP, pD3, pregnenolone or ethanol vehicle (control) and luciferase activity was measured in cell extracts. D and E. Inhibition of cell growth as measured by sulforhodamine b assay (protein content) after 72 h of treatment of non-pigmented (D) and pigmented (E) PIG1 human melanocytes. Inserts show the color of representative cell pellets. Significant differences versus ethanol-treated control cells were defined as *p<0.05, **p<0.01, ***p<0.001.

**Figure 12:**
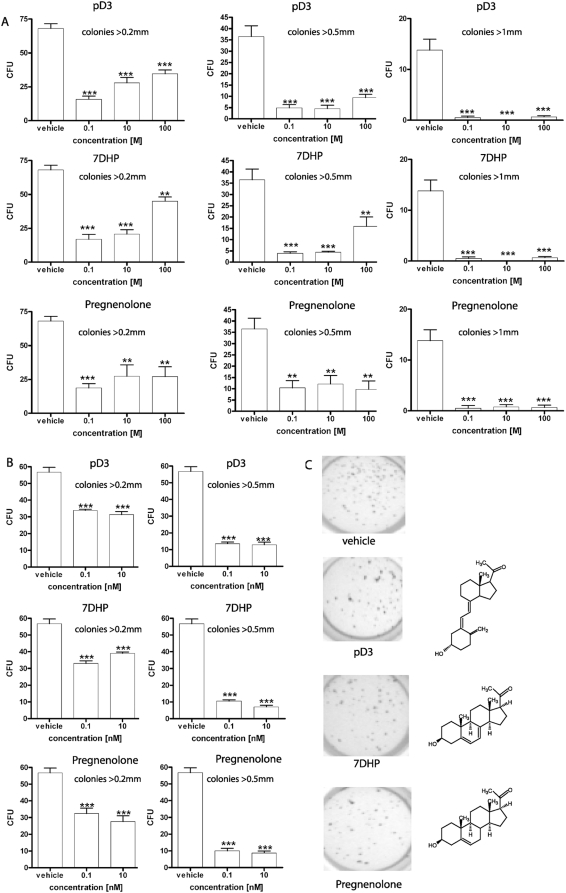
7DHP, 20-oxopregnacalciferol (pD3) and pregnenolone inhibit growth of melanoma cells in soft agar. A: hamster AbC1 melanoma; B: human SKMEL-188 melanoma; C: visualization of SKMEL-188 colonies by MTT reagent. Decreased number and size of colonies was observed in both cell lines. Significant differences *versus* ethanol-treated cells were defined as *p<0.05, **p<0.01, ***p<0.001.

Having documented that the adrenal gland can transform 7DHC to 7DHP, we tested the biological activity of 7DHP, pD3 (vitamin D3-like product of UVB-mediated photoconversion of 7DHP [37]) and pregnenolone as a control, in cultured normal and malignant skin cells (Figs. 11, 12). Specifically, the above compounds inhibited in a dose-dependent fashion proliferation of epidermal HaCaT keratinocytes and immortalized normal epidermal melanocytes (PIG1; pigmented and nonpigmented cultures)(Fig. 11). There was no significant difference between the effects of 7DHP and pregnenolone, however, pD3 was less potent than the above compounds. Furthermore, 7DHP, pregnenolone, pD3 and 1,25(OH)2D3 inhibited NFκB activity in HaCaT keratinocyes, with 7DHP being the most efficient inhibitor, which again was significantly more potent than pD3 (Fig. 11B). Next we tested their anti-cancer activity against hamster AbC1 and human SKMEL-188 melanoma cells. Here all of the three compounds showed similar capacity to inhibit growth of melanoma cells in soft agar and the effect was already significant at doses as low as 0.1 nM (Fig. 12). Thus, products of P450scc mediated metabolism such as 7DHP and pregnenolone are biologically active in both normal and malignant cells of epidermal origin. This raises the possibility that they may act in auto or paracrine fashion after local production [10], [19]. Interestingly, pD3 (vitamin D3-like photoderivative of 7DHP) also demonstrates a potent anti-proliferative potential, but is less active than its precursor 7-DHP. Nevertheless, this biological activity has significant implications taking into consideration that pregnacalciferol and 20-oxopregnacalciferol have no or very low calcemic activity, respectively [37], [38]. Furthermore, since the skin is exposed to solar radiation during the daytime, 7DHP (whether delivered by the circulation or produced locally) will be converted to 20-oxopregnacalciferol after absorption of UVB photons. Therefore, we tested whether the mammalian skin (a major site of 7DHC accumulation in the body) has the potential to transform 7DHC to 7DHP and further metabolize it in a similar manner to the adrenal gland.

### 4. Metabolism of DHC and 7DHP in skin extracts

**Figure 13:**
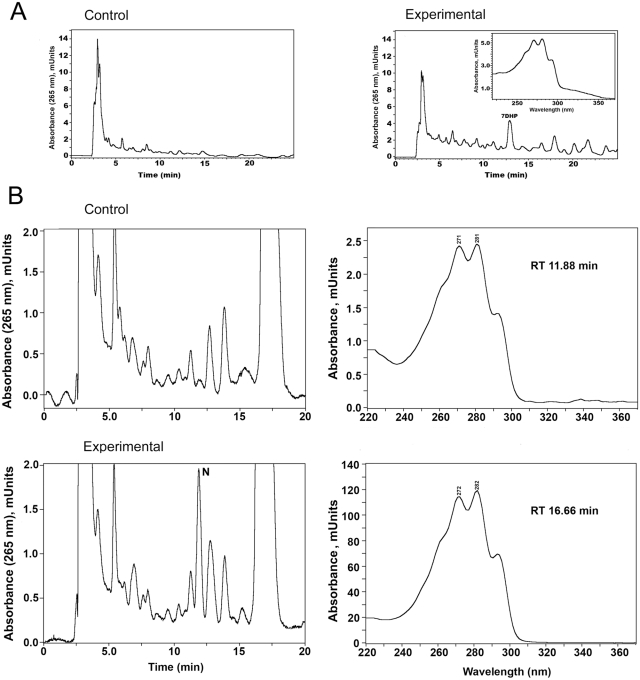
Metabolism of 7DHC and 7DHP by rat skin. A. To study the skin capability to transform 7DHC to 7DHP isolated mitochondria were incubated with 7DHC (0.5 mM) in the presence (experimental) or absence (control) of isocitrate and NADPH. 7DHP: product with the same retention time and UV spectra (inset) as 7DHP standard. B. Incubation of crude skin extract with 7DHP substrate (RT 16.7 min) in the presence of isocitrate and NADPH (experimental) resulted in appearance of a 5,7-diene product (N) with RT 11.9 min, which was absent in control reaction mixture (isocitrate and NADPH were not added) (control).

Previously we have reported the expression of P450scc and its electron transfer partners in the skin and demonstrated the capability of skin mitochondria and intact melanoma cells to transform cholesterol to pregnenolone [10]. Since the activity of P450scc is low in skin [10] (too low to detect ex-vivo production of 7DHP by above methods), we isolated mitochondria from rat skin to test 7DHC metabolism. Fig. 13A clearly shows that 7DHC is metabolized to 7DHP, based on the identical RT of the product to authentic standard and the expected 5,7-diene UV spectrum. We were also able to show that the skin extracts could metabolize 7DHP to a more polar 5,7-diene product with RT of 11.9 min (Fig. 13B). This indicates that the skin has a potential to transform 7DHC to 7 DHP and then further modify the 7DHP (most likely by hydroxylation). Because of the noisy background in the chromatogram for the above experiment using skin mitochondria, we isolated a skin microsomal fraction and incubated it with 7DHP, then analyzed the products on an LC-MS QP8000a as detailed in Materials and Methods. We have found that skin microsomes metabolized 7DHP to two new products with UV λmax 280 indicating modification of the A and B rings with likely generation of 4,6-dienes (not shown). Although the structure of these compounds and enzymes involved in their synthesis remain to be identified, we can clearly conclude that the skin has the capability to produce 7DHP and to metabolize it with preservation of the 5,7-dienal structure or at least its degree of unsaturation. Such a capability supports an auto or paracrine role for 7DHP itself or of its metabolites including pregnacalciferols generated photolytically.

### 5. Conclusions and Perspective

Following our previous demonstration that mammalian P450scc in an *in vitro* reconstituted system can cleave the side chain of 7DHC to produce 7DHP [10], we now demonstrate that adrenal glands from different mammalian species maintained ex vivo can efficiently transform 7DHC to 7DHP in a process that is dependent on P450scc activity and that includes generation of 22(OH)7DHC and 20,22(OH)_2_7DHC as intermediates. Furthermore, 7DHP can be metabolized further along the Δ^4^ and Δ^5^ steroidogenic pathways with production of 7-dehydroprogesterone and 17(OH)7DHP as intermediates, which depends on the mammalian species. Thus, normal adrenal glands can produce steroidal 5,7 and 4,7-dienes from 7DHC via known steroidogenic enzymes, a capability previously thought to be restricted to pathological conditions (SLOS secondary to genetic inactivation of 7Δ reductase [17], [18], [32], [33]) or to mare's gonads under physiological conditions for the synthesis of equilin (delta-7-estrone) [39].

The skin is known to express functional steroidogenic enzymes and skin cells or hair follicles can produce steroidal intermediates, corticosterone or cortisol [10], [19], [40]–[44]. Therefore, although the activity of this pathway is comparatively low [10], it is not surprising that skin mitochondria can produce 7DHP, while skin extracts or isolated microsomes can, further metabolize it to steroidal 5,7-dienes or to products with a modified B ring structure. Such a capability is highly significant because the skin is an important repository of 7DHC (making it readily available to cutaneous P450scc), and because its exposure to solar radiation can cause the unsaturated B ring of 7-DHP and its hydroxyderivatives to break generating novel vitamin D-like compounds without the side chain [45], [46], in a process similar to production of vitamin D3 [21]. This significance is further enhanced by our demonstration that pregnenolone, 7DHP and pD3 display different biological activities on skin cells. Thus, the described above new novel steroido/secosteroidogenic pathway could in the skin potentially operate in an intra-, auto- or paracrine mode.

In summary, we define a novel steroidogenic pathway: 7DHC→22(OH)7DHC→20,22(OH)_2_7DHC→7DHP, with a potential for further 7DHP metabolism mediated by other steroidogenic enzymes including 3βHSD or CYP17(depending on mammalian species). The potential flux through the pathway and biological activity of its products depends on the organ (adrenals *vs* skin), and of which 5,7-dienal intermediates can be converted to active secosteroids after exposure to solar radiation.

## Materials and Methods

### Tissue collection and preparation

Adrenal glands were obtained from 2–4 months old male and female rats (Wistar or Sprague-Dawley), after killing under anesthesia as described previously [10], [47]. In addition, adrenal glands were obtained from pigs (York-Land, 3 months old males and females), rabbits (New Zeland, 3–5 months old males and females) and dogs (Walker hounds, 6 months old males). The Institutional Animal Care and Use Committee at UTHSC approved the original protocols; similarly the experiments were approved by the Belarus University Animal Care and Use Committee. All animal experimentation described was conducted in accord with accepted standards of human animal care, as outlined in the ethical guidelines.

### Metabolism of steroids by adrenal glands

Fresh adrenal glands were dissected from connective tissue, cut with scissors into small fragments and suspended in buffer (pH 7.4) containing 33 mM Tris aminomethane, 110 mM NaCl, 5 mM KCl, 2.5 mM CaCl_2_, 1 mM MgSO_4_, 1 mM KH_2_PO_4_ and 2 mg/ml glucose (3). Prior to assay, adrenal fragments were pre-incubated in 0.5 ml of the above buffer (50–100 mg of adrenal tissue per ml) in a water bath for 5 min at 37°C with continuous gentle shaking. Reactions were started by adding isocitrate (final concentration 5 mM) followed by addition of 7DHC or 7DHP (final concentration of 0.5 mM) and tubes incubated at 37°C for 4–6 h or as indicated. Five or 10 mM stock solutions of 7-dehydrocholesterol (cholesta-5,7-dien-3β-ol; 7DHC) or 7-dehydropregnenolone (3β-hydroxypregna-5,7-dien-20-one; 7DHP) were prepared in 45% 2-hydroxypropyl-β-cyclodextrin immediately before use. 7DHC was purchased from Sigma Chemical Co. (St. Louis, MO), while 7DHP was synthesized and purified as described [1]. The initial testing for time- and dose- dependent effects showed that the above tissue and substrate concentrations, and time of incubation were optimal for metabolism of 7DHC and 7DHP (not shown). In control experiments, either 7DHC or 7DHP were omitted from the incubation mixture or fragments of adrenal glands were boiled for 5 min before addition of the substrates. The reactions were stopped by placing the tubes on ice and steroids were extracted twice with methylene chloride and dried under nitrogen or by using a rotational vacuum concentrator RVC 2–18 (Christ, Germany). The residues were re-dissolved in methanol for further analysis.

### Reverse phase-liquid chromatography (RP-HPLC) and mass spectrometry analyses

Three different HPLC and mass spectrometry systems were used due to the project being carried out at several separate institutions. The independent analyses in the different laboratories confirmed the major findings of this study. First, RP-HPLC was performed using a dual pump chromatography system (Waters Ass. Inst) equipped with a Waters Atlantis dC18 column (100×4.6 mm, 5 µm particle size) using a mobile phase of methanol in H_2_O, as described previously [48]. In most experiments, a gradient of methanol in water (55%–97%) was used for elution at a flow rate 1 mL/min (42 min), followed by a wash with 97% methanol (8 min), unless specified in the figures. Fractions were monitored with a photodiode array or dual wave-length detector. Alternatively, samples were resolved by a Waters HPLC system as described above, but the separation was accelerated by running the gradient (55%–97%) for 20 minutes followed by the 97% methanol wash for 7 min. Fractions were collected every 30 seconds and analyzed with a Bruker Esquire-LC/MS Spectrometer by direct injection into the MS module [45].

Mass spectra were also recorded using a Bruker Esquire-LC/MS Spectrometer equipped with an electrospray ionization (ESI) source, a Waters Atlantis dC18 column (100×4.6 mm, 5 µm particle size), using a mobile phase of 85% methanol and 15% water at a flow rate of 0.25 mL/min (15 min), as described previously [45].

Some samples were analyzed by HPLC on an LC–MS QP8000a (Shimadzu, Japan) equipped with a Restec Allure C18 column (150×4.6 mm; 5 µm particle size; 60 Å pore size), UV/VIS photodiode array detector (SPD-M10Avp) and single quadrupole mass-spectrometric detectors [47], [49]. The LC workstation CLASS-8000 software was used for system control and data acquisition (Shimadzu). The mass spectrometer was operated in the atmospheric pressure chemical ionization (APCI) mode; positive ion mode was used with nitrogen as the nebulizing gas. The MS parameters were as follows: nebulizer gas flow rate 2.5 L·min^−1^; probe high voltage 4.5 kV; probe temperature 480°C; curved desolvation line heater temperature 285°C. Analyses were carried out in the scan mode from *m/z* 280–450. For minced adrenal glands and skin microsome incubations, elution of samples was carried out using a linear gradient at a flow rate of 0.5 mL·min^−1^ and temperature of 40°C, from 65 to 70% of methanol and 0.1%(v/v) acetic acid (0–15 min); from 70 to 85% of methanol and 0.1%(v/v) acetic acid (15–30 min); from 85 to 100% of methanol and 0.1%(v/v) acetic acid (30–35 min); followed by isocratic elution with 100% of methanol and 0.1%(v/v) acetic acid from 35 to 45 min. The elution for samples of skin mitochondria was carried out isocratically from 0–12 min (80% methanol and 0.1% acetic acid, followed by a linear gradient from 80–100% methanol and 0.1% acetic acid (12–20 min), then isocratically using 10% methanol and 0.1% acetic acid from 20–60 min. For skin extracts the mobile phase consisted of 75% methanol and 0.1% acetic acid.

### Metabolism of 7DHC and 7DHP by rat skin

Skin extracts, microsomes and mitochondria were prepared from the shaved full thickness dorsal skin, after removal of the subcutaneous fatty tissue. Skin was minced thoroughly with surgical scissors and a tissue grinder, placed in isolation buffer comprising 2 mM HEPES (pH 7.4), 70 mM sucrose, 220 mM D-mannitol and 2 mM EDTA then homogenized using a glass pestle. For the isolation of mitochondria and microsomes the homogenate was centrifuged at 576 g for 10 min at 4°C and the resulting supernatant was removed and centrifuged again at 10,000 g for 20 min at 4°C to get a mitochondrial fraction [40]. The supernatant was used for the isolation of microsomes by centrifuging at 105,000 *g* for 1 h at 4°C. The mitochondrial pellet was re-suspended in isolation buffer and the centrifugation was repeated twice under the same conditions [10], [49]. The microsomal pellet was re-suspended in 10 mM Tris-HCl (pH 7,4) and the centrifugation was repeated at 105,000 *g* for 1 h at 4°C. The washed microsomal fraction was re-suspended in 10 mM Tris-HCl (pH 7,4) and immediately used for the enzyme assay [50]. To study 7DHP metabolism in skin homogenate, the homogenate was centrifuged at 1200 *g* at 4°C for 10 min and the supernatant was collected and used for further experiments.

To test for metabolism of 7DHC, the washed mitochondrial fraction was re-suspended in HEPES-buffered medium (0,25 M sucrose, 50 mM HEPES, 20 mM KCl, 5 mM MgSO_4_, 0.2 mM EDTA, (pH 7.4)) and incubated with 0.5 mM 7DHC and 5 mM isocitrate at 37°C for 4 h. The control comprised mitochondria boiled prior to the incubation. Reactions were stopped by adding methylene chloride, and samples extracted twice with this solvent. The methylene chloride layers were combined and dried in a rotational vacuum concentrator RVC 2–18 (Christ, Germany). The residues were re-dissolved in methanol and subjected to HPLC analysis on an LC–MS QP8000a (Shimadzu, Japan). To study 7DHP metabolism by skin homogenate, the 1200 *g* supernatants were pre-incubated for 10 min at 37°C with 20 µM 7DHP (added form a methanol stock) in 0.5 ml of 10 mM Tris-HCl, (pH 7.4). Reactions were started by adding 5 mM isocitrate and 0.5 mM NADPH and samples incubated at 37°C for 90 min. Reactions were then stopped and analyzed by HPLC as above. To study microsomal metabolism the skin microsomes were pre-incubated in 10 mM Tris-HCl (pH 7.4) with 0.5 mM 7DHP for 10 min at 37°C with continuous gentle shaking. Reactions were initiated by adding 1 mM NADPH and carried out at 37°C for 2 h.

### Interaction of 7DHC and 7DHP with purified steroidogenic enzymes

Transfer of 7DHC and cholesterol from acidic dioleoyl phosphatidylcholine donor vesicles containing cardiolipin, to neutral dioleoyl phosphatidylcholine acceptor vesicles, was measured in the presence and absence of N-62 StAR protein (a gift from Walter Miller, University of California, San Francisco). The incubations and subsequent separation of acidic and neutral vesicles by DEAE Sepharose chromatography, were done as described before [51].

Cytochrome P450scc was purified from bovine adrenals as before [52]. The side chain cleavage activity of P450scc in phospholipid vesicles was determined using [4-^14^C]cholesterol as substrate. Vesicles were prepared from dioleoyl phosphatidylcholine and cardiolipin in the molar ratio of 85∶15, plus tracer [4–14C]cholesterol, using a bath sonicator as before [51]. When required, 7DHC was included in the vesicles at a molar ratio to phospholipid of 0.2. Incubations with P450scc were carried out for 2 min at 37°C, the reaction stopped and steroids extracted with dichloromethane as described previously [51]. Radiolabelled cholesterol and pregnenolone were separated by thin-layer chromatography on silica gel G with three developments in hexane:acetone (7∶3, v/v). The areas of silica gel corresponding to cholesterol and pregnenolone were removed separately and the associated radioactivity determined by scintillation counting.

3βHSD was partially purified from 114 g human placenta by extraction of a combined mitochondrial and microsomal fraction with 0.6% sodium cholate, followed by chromatography on DEAE Sepharose as described by Thomas et al [53]. Activity was assayed at 35°C from NAD^+^ reduction monitored at 340 nm in an incubation mixture comprising 3βHSD (2.25 µg/mL), 20 mM potassium phosphate pH 7.5, 0.1 mM EDTA, 0.1 mM dithiothreitol 0.1 mM NAD^+^, 0.4% Emulgen 913 and 20% glycerol. Substrates (5–30 µM) were added in ethanol to a final ethanol concentration of 2%.

### Cell culture experiments

Immortalized human keratinocytes (HaCaT) were cultured in Dulbecco's Modified Eagle Medium supplemented with glucose, L-glutamine, pyridoxine hydrochloride (Cell Grow), 5% foetal bovine serum (FBS) and 1% penicillin/streptomycin/amphotericin antibiotic solution (Sigma) [54]. The melanoma cell lines, human SK Mel 188 and hamster AbC1, were grown in F10 media (Gibco) supplemented with 5% FBS [55]. Human immortalized melanocytes PIG1, including a pigmented line (gift of Dr LePoole [56]) and an amelanotic variant (that lost the ability to produce pigment during subculturing [57]), were grown in medium 254 supplemented with 5%HMGS for melanocytes from Cascade Biologics.

To study DNA synthesis, HaCaT keratinocytes were plated in 24-well plates at 50,000 cells/well. After overnight incubation, pD3, 7DHP and pregnenolone, initially dissolved in ethanol before dilution with DMEM medium containing 5% charcoal-treated serum, were added to the culture medium at the concentrations listed (see Results). After 44 h, [^3^H]-thymidine (specific activity 88.0 Ci/mmol; Amersham Biosciences, Picataway, NY, USA) was added to a final concentration of 1 µCi/mL medium. After 4 h, media were discarded, cells washed with cold PBS and incubated in 10% TCA for 30 min. Cells were washed again with PBS and incubated with 1 N NaOH (100 µL/well) for 30 min at 30°C. The extracts were collected, scintillation cocktail added and ^3^H-radioactivity was measured with a beta counter (Direct Beta-Counter Matrix 9600; Packard), as described previously [12], [55].

MTT [3-(4,5-dimethylthiazol-2-yl)-2,5-diphenyltetrazolium bromide] viability tests were performed as previously described [54]. HaCaT cells were seeded at 5,000 per well in 96 well plates. After 12 h media were changed and pD3, 7DHP and pregnenolone were added to achieve test concentrations 10^−8^–10^−10^ M. After 68 h of incubation, MTT assays were performed as described previously with absorbance measured at 570 nm [26], [54].

To measure cell growth an additional sulphorhodamine b (SRB) assay, which estimates the protein content of cells, was performed [58]. Briefly, immortalized human epidermal melanocytes (line PIG1) were seeded at 10,000 cells per well in 96 well-plates with melanocyte growth medium [40]. After 12 h the medium was changed to medium containing pD3, 7DHP and pregnenolone. After 71 h of incubation, 50% acetic acid was added to a final concentration of 20%, cells stained with SRB (Sigma), washed with 1% acetic acid, dried, dissolved in Tris-HCl and the absorbance measured at 565 nm [58].

The tumorogenicity of SKMEL-188 and AbC1 melanoma cells was determined by assaying their ability to form colonies in soft agar. Cells growing in monolayer culture were trypsinized and re-suspended (1,000 cells/well) in 0.25 ml medium containing 0.4% agarose and 5% charcoal-stripped serum (HyClone). Cell suspensions were added to a 0.8% agar layer in 24 well plates. pD3, 7DHP and pregnenolone were added from ethanol stocks to final concentrations ranging from 0.1 nM to 100 nM. An ethanol solvent control was included in the assay. Soft agar colonies were scored and stained after two weeks, with 0.5 mg/ml MTT reagent (Promega). Colonies were counted under the microscope and the number of units was calculated from the number of colonies formed divided by the number of cells seeded ×100.

To measure the NFκB activity, HaCaT keratinocytes were transfected with the NFκB-Luc construct using Lipofectamine Plus (Invitrogen, Carlsbad, CA) as described previously [59]. Twenty four hours after transfection the media were changed and pD3, 7DHP, pregnenolone or ethanol (vehicle control) were added and cells incubated for 24 h. The firefly luciferase and Renilla luciferase signals were recorded with a TD-20/20 luminometer (Turner Designs, Sunnyvale, CA) and and activities calculated as described previously [57], [59].

Statistical analysis was performed with GraphPad Prism Version 4.0 (GraphPad Software Inc., San Diego, CA, USA) using t test. Differences were considered significant when p<0.05.
